# Leveraging Shapley Additive Explanations for Feature Selection in Ensemble Models for Diabetes Prediction

**DOI:** 10.3390/bioengineering11121215

**Published:** 2024-11-30

**Authors:** Prasant Kumar Mohanty, Sharmila Anand John Francis, Rabindra Kumar Barik, Diptendu Sinha Roy, Manob Jyoti Saikia

**Affiliations:** 1 Department of Computer Science and Engineering, National Institute of Technology, Meghalaya 793003, India; 2 Department of Computer Science, King Khalid University, Abha Campus, Rijal Alma, Abha 61421, Saudi Arabia; 3 School of Computer Applications, KIIT Deemed to be University, Bhubaneswar 751024, India; 4 Biomedical Sensors & Systems Lab, University of Memphis, Memphis, TN 38152, USA; 5 Electrical and Computer Engineering Department, University of Memphis, Memphis, TN 38152, USA

**Keywords:** diabetes prediction, influential feature values, ensemble models, Shapley additive explanations

## Abstract

Diabetes, a significant global health crisis, is primarily driven in India by unhealthy diets and sedentary lifestyles, with rapid urbanization amplifying these effects through convenience-oriented living and limited physical activity opportunities, underscoring the need for advanced preventative strategies and technology for effective management. This study integrates Shapley Additive explanations (SHAPs) into ensemble machine learning models to improve the accuracy and efficiency of diabetes predictions. By identifying the most influential features using SHAP, this study examined their role in maintaining high predictive performance while minimizing computational demands. The impact of feature selection on model accuracy was assessed across ten models using three feature sets: all features, the top three influential features, and all except these top three. Models focusing on the top three features achieved superior performance, with the ensemble model attaining a better performance in most of the metrics, outperforming comparable approaches. Notably, excluding these features led to a significant decline in performance, reinforcing their critical influence. These findings validate the effectiveness of targeted feature selection for efficient and robust clinical applications.

## 1. Introduction

Diabetes is a global health crisis, significantly impacting India as well. Characterized by its chronic nature and high prevalence, diabetes affects over 537 million people worldwide, which is expected to exponentially increase to 783 million by 2045 [1]. While diabetes can be accurately diagnosed through blood tests, this work is not intended to replace these established diagnostic methods. Instead, it addresses a complementary yet critical dimension of diabetes management and prevention: early prediction and risk assessment [2,3,4]. Particularly for individuals in prediabetic stages or those at high risk of developing the condition, those who receive an alert can take precautionary actions to avoid it. The disease’s severity is underscored by its complications, such as cardiovascular disease, kidney failure, and increased mortality, which impose a significant economic burden on healthcare systems [5]. For instance, the management of cardiovascular complications alone accounts for a significant portion of diabetes-related healthcare costs. At the same time, the treatment of kidney failure often requires expensive interventions like dialysis or transplants. Early prediction and management are crucial; however, challenges such as inadequate healthcare resources and low awareness, particularly in rural areas, hinder effective management and escalate the urgency for enhanced educational and preventive measures [6]. The research underscores the importance of technological advancements in diabetes prediction to mitigate these impacts, advocating for improved healthcare strategies and policies tailored to global and local contexts [7].

In recent years, the rise of Explainable AI (XAI) has revolutionized various domains, including healthcare, by enhancing transparency and fostering trust in automated decision-making systems. This is particularly crucial in diabetes prediction, where understanding the reasoning behind AI-driven insights can improve clinical decision-making and patient outcomes. Shapley Additive Feature (SAF) explanations have emerged as a vital tool in this context, providing quantitative insights into the contributions of individual features toward model predictions.

Presenting a systematic analysis to study the nature of SAF in diabetes data and developing methods to handle these features is crucial for several reasons. Firstly, SAF enhances the interpretability of predictive models by quantifying each feature’s contribution to predictions, which is critical in complex medical decisions like diabetes management. This clarity supports healthcare providers in understanding and trusting AI-driven decisions. Secondly, analyzing SAF can improve model accuracy by identifying the most impactful features, thus refining the models for better performance. Thirdly, a detailed analysis ensures fairness and ethical considerations are met, preventing models from relying on biased or irrelevant features. However, SAF has certain limitations, such as computational complexity, particularly when applied to large datasets, and while assembling different models, each should have the same feature importance rankings. Understanding feature contributions through SAF enables tailored medical interventions, potentially improving patient outcomes by allowing more personalized treatment plans.

This research explores the impact of different types of features, such as the top three Shapley influencing features and other features excluding these top features in diabetes prediction, using machine learning (ML) algorithms. It uniquely analyzes highly influential and less contributing feature sets to understand their roles in various ML frameworks. By conducting thorough and systematic experiments, the study compares the effectiveness of these feature sets individually and combined. Additionally, it evaluates multiple ML techniques, including Gradient Boost, Extreme Gradient Boost, AdaBoost, CatBoost, logistic regression, decision trees, random forests, artificial neural networks, and support vector machines. A framework for ensemble classification models is also implemented, enhancing the prediction accuracy by integrating various classification methods. This comprehensive approach allows a deep understanding of how different feature sets and ML models affect diabetes prediction accuracy.

The major contributions of this work can be summarized as follows:

- The research evaluates the effectiveness of two distinct feature sets—highly influential and less contributing features—in diabetes prediction, leveraging Shapley Additive explanations for feature selection.
- Experimental results demonstrate that adopting the top three highly influential features significantly optimizes the performance of machine learning models, achieving better performance in most of the metrics with ensemble models, which is comparable to using all features while improving computational efficiency.
- This study conducts a comprehensive analysis of ten diverse machine learning algorithms using individual and combined feature sets, offering a detailed comparison across multiple performance metrics.
- The work demonstrates a practical and effective approach for targeted feature selection, contributing to efficient deployment of predictive models in clinical settings, where quick and accurate decision-making is critical.

This article is structured as follows: Section 2 briefly describes several disease prediction or prevention approaches with the help of AI or XAI, followed by their gaps. Section 3 provides a detailed statistical data analysis of all the features and a complete description of the diabetes dataset. Section 4 describes the methodology, the machine learning algorithms employed, and the criteria for feature categorization into highly influential and less contributing sets. Section 5 presents the experiments’ results, which assess the effectiveness of different feature sets across multiple ML algorithms. Section 6 discusses these findings, interpreting the implications for diabetes prediction and the potential for clinical application. The complete work is summarized, and future work is outlined in Section 7. This structured approach ensures a comprehensive exploration of the role of machine learning in enhancing diabetes prediction, with a particular focus on the impact of feature selection.

## 2. Literature Survey

Recent technological advancements have significantly improved the prediction of critical diseases like diabetes. Advanced machine learning and ensemble learning techniques are now widely used to predict diabetes more accurately. These approaches leverage large datasets to identify patterns and predict diabetes risk effectively. Ensemble learning, particularly boosting and gradient boosting, has shown high accuracy in predicting diabetes by analyzing clinical parameters and improving feature selection processes [8]. In 2022, El Massari et al. [9] conducted a comprehensive study comparing various machine learning algorithms, including SVM, KNN, ANN, Naive Bayes, logistic regression, and decision trees, specifically for diabetes prediction. The researchers highlighted the superior performance of ontology-based classifiers and SVM, suggesting that these models are particularly effective in handling the complexities of medical diagnostic data.

Recent studies highlight advancements in diabetes prediction models. Belsti et al. identified CatBoost as the optimal classifier with a 93% AUC for gestational diabetes, surpassing traditional methods [10]. Similarly, Febrian et al. found Naive Bayes more effective than KNN for diabetes prediction using health attributes [11].

Another article [12] explored how artificial intelligence and machine learning could revolutionize diabetes management, particularly in enhancing cardiovascular care. It critically assessed the potential of AI-driven tools for diagnosis, risk assessment, and personalized diabetes treatment while addressing challenges such as bias and regulatory considerations in healthcare applications. A novel diabetes prediction model was introduced in [13], leveraging Boruta feature selection and ensemble learning, including K-Means++ clustering for data segmentation and stacked classifiers for improved predictive accuracy. Another research work [14], evaluated on the PIMA dataset, achieved 90.57% accuracy on a cloud-based environment, outperforming state-of-the-art models with fewer features. Similarly, Dashdondov et al. [15], Liu et al. [16] used machine learning models, whereas Chang et al. [17], Priyadarshini et al. [18], Chou et al. [19], Dutta et al. [20] performed diabetes prediction using ensemble techniques.

In recent years, Explainable AI has been increasingly integrated into diabetes prediction systems. Tasin et al. [21] employed XAI techniques to enhance prediction accuracy and interpretability, facilitating practical applications through a web and mobile interface for real-time diabetes prediction. Similarly, Wang et al. [22] used LIME to simplify artificial neural networks into intuitive CART models, improving interpretability for type 2 diabetes diagnosis. Lalithadevi et al. [23] utilized a random forest model with SHAP explainability to accurately predict diabetic retinopathy risk in type II diabetes, offering insights into key socio-clinical predictors. A self-explanatory interface for diagnosing diabetes using SHAP-based local explanations is introduced in [24], showcasing transparent predictions to improve patient outcomes. A framework for early prediction of Gestational Diabetes Mellitus (GDM) using a novel data methodology and an explainable deep neural network algorithm is proposed in [25], offering high accuracy and interpretability.

While significant advancements have been made in diabetes prediction using machine learning and XAI, there remains a gap in integrating Shapley Additive Features explanations into ensemble models to balance interpretability, computational efficiency, and model accuracy.

## 3. Dataset Analysis

The dataset employed in this study originates from the National Institute of Diabetes and Digestive and Kidney Diseases and is designed to assist in diagnosing diabetes among patients. This dataset, meticulously selected from a more extensive database, comprises female patients from the Pima Indian community, all aged 21 years or older [26]. It incorporates a range of medical predictor variables, including the number of pregnancies, Body Mass Index (BMI), insulin levels, and age, along with a target variable named ‘Outcome’. The selection of these variables is informed by their relevance in predicting the onset of diabetes, making the dataset a crucial tool in advancing diabetes research. This targeted compilation of data enriches the research article and significantly contributes to the scientific community’s efforts in understanding and diagnosing diabetes more effectively.

Table 1 provides detailed attribute information for the diabetes dataset, including descriptions of 768 patients and ranges for each variable. Attributes range from the number of pregnancies to glucose levels, insulin measurements, BMI, and more. Each variable is crucial for diagnostically predicting diabetes, with the target variable ‘Outcome’ indicating the presence or absence of diabetes.

Table 2 provides a comprehensive summary of the attributes within the diabetes dataset, encompassing 768 observations. It details key statistical metrics for each attribute, including the count, mean value, standard deviation, minimum and maximum values, and quartiles (Q1, Q2, Q3) of the attributes analyzed.

## 4. Predictive Modeling Framework

**Figure 1:**
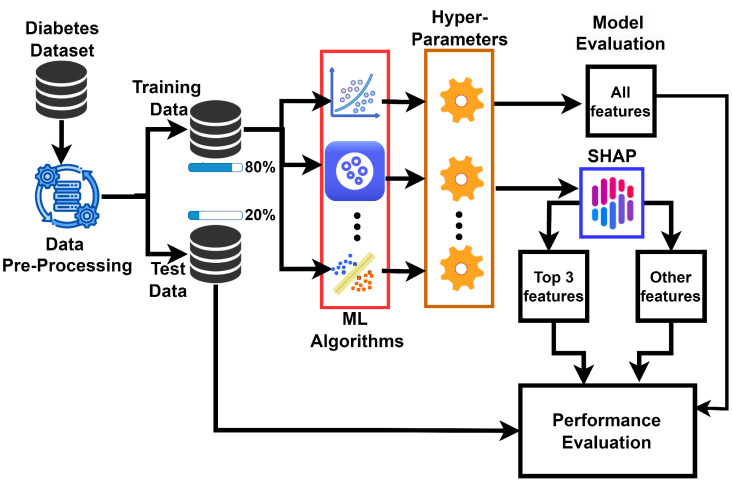
System model for prediction of diabetes with multiple learning models.

Benchmark machine learning frameworks have been adopted in this work to conduct a comparative study on several medical features for diabetes prediction. The selection of frameworks is informed by intuition drawn from prior successful implementations in similar applications, and each model is analyzed for its effectiveness in structured datasets typical in medical records. Commonly used classifiers such as artificial neural networks (ANNs), support vector machines (SVMs), and random forest have been recognized in prior research for their applicability in systems predicting diabetes. Consequently, ten machine learning algorithms have been employed. A SHAP summary plot is generated for each model to categorize the dataset features into two sets: the top three most influential features and the remaining features. Performance evaluations for each category are conducted using several metrics, and these performances are compared with those obtained using all features. The best-performing model will be identified based on different performance metrics. The modeling framework is presented in Figure 1. The aforementioned learning models are briefly described as follows.

### 4.1. Gradient Boosting and Extreme Gradient Boosting (XGBoost)

Gradient Boosting [27] is an ensemble learning technique that builds a sequence of weak learners, typically decision trees, in an iterative manner. Each tree in the sequence is trained to correct the residual errors from the previous trees. Gradient Boosting is highly effective for complex predictive tasks such as diabetes disease prediction, where it can model non-linear relationships and interactions among medical features. The prediction function is given by:

(1) $$\hat{y}=\sum_{n=1}^{N}\Gamma_{n}h_{n}\left(x\right)$$

where $h_{n}\left(x\right)$ is the *n*-th decision tree, $\Gamma_{n}$ is the weight assigned to that tree, and *N* is the total number of trees in the model. Each subsequent tree is trained on the residuals of the previous trees, improving the overall model accuracy in predicting diabetes risk.

Extreme Gradient Boosting (XGBoost) is an enhanced version of Gradient Boosting that incorporates regularization techniques to prevent overfitting, second-order gradients to improve optimization, and a tree-pruning mechanism to speed up the training process. These enhancements make XGBoost particularly effective for large, high-dimensional datasets typical in medical applications, including diabetes prediction. XGBoost can handle missing data and is highly scalable, efficiently processing medical records with thousands of patient features.

### 4.2. AdaBoost and CatBoost

AdaBoost (Adaptive Boosting) is an ensemble method where multiple weak learners, such as shallow decision trees, are trained sequentially. Instead of focusing on residuals like Gradient Boosting, AdaBoost adapts the weights of training instances, giving more importance to previously misclassified samples. This mechanism ensures that the model focuses on hard-to-predict cases, which is crucial for improving the classification of patients with complex or borderline diabetic conditions. The prediction function is expressed as:

(2) $$F\left(x\right)=\sum_{t=1}^{T}\alpha_{t}h_{t}\left(x\right)$$

where $\alpha_{t}$ represents the weight assigned to the *t*-th weak classifier, and $h_{t}\left(x\right)$ is the weak classifier itself. AdaBoost effectively improves classification performance for diabetes prediction, but it may be less robust when the dataset contains noise.

CatBoost, on the other hand, is specifically designed to handle datasets with categorical features, making it particularly suitable for medical datasets that include patient demographics, medical history, and lifestyle factors, all of which play significant roles in predicting diabetes risk. CatBoost’s unique algorithm processes categorical data efficiently by employing ordered boosting and avoiding the need for extensive one-hot encoding, which can otherwise lead to performance degradation. This feature allows CatBoost to achieve high accuracy while maintaining computational efficiency, making it ideal for diabetes prediction tasks where numerical and categorical data are crucial for accurate predictions.

### 4.3. LightGBM

It is a gradient-boosting framework [28], which is particularly effective for large and sparse medical datasets often seen in diabetes studies. This model enhances speed and efficiency through histogram-based optimizations, making it suitable for real-time diabetes prediction applications. The model equation is:

(3) $$\hat{y}=\sum_{i=1}^{M}\gamma_{i}h_{i}\left(x\right)$$

where $h_{i}\left(x\right)$ represents the i-th tree, and $\gamma_{i}$ are the tree weights.

### 4.4. Support Vector Machine (SVM) and Artificial Neural Network (ANN)

For diabetes detection, SVM is used to form a hyperplane that efficiently classifies [29] diabetic and non-diabetic patients by:

(4) y=w·x+b

Meanwhile, an ANN is employed to model complex non-linear relationships indicative [30] of diabetes patterns in medical data:

(5) y=σ(w·x+b)

where σ is the activation function.

### 4.5. Decision Tree, Random Forest, and Logistic Regression

These models are instrumental in diabetes detection. Logistic regression estimates the probability [30] of diabetes as follows:

(6) $$P\left(y=1|x\right)=\frac{1}{1+e^{-(w\cdot x+b)}}$$

Random forest, an ensemble of decision trees, is used to mitigate overfitting and improve the predictive reliability for diabetes diagnosis.

### 4.6. Shapley Additive Explanations

Shapley Additive explanations (SHAPs) are a powerful interpretability tool [31] derived from cooperative game theory, particularly the Shapley values, which are used to determine the contribution of each player (or feature) to the overall game (or prediction model). In diabetes detection, SHAP values are instrumental in understanding how each dataset feature influences the prediction outcome, thus providing clear insights into which factors are most predictive of diabetes. The SHAP value for each feature is calculated as follows:

(7) $$\phi_{j}=\sum_{S\subseteq N\setminus \{j\}}\frac{|S|!(|N|-|S|-1)!}{|N|!}\left[f_{x}\left(S\cup \left\{j\right\}\right)-f_{x}\left(S\right)\right]$$

In the above:

- $\phi_{j}$ is the SHAP value for feature *j*;
- *S* is a subset of features excluding *j*;
- *N* is the total set of features;
- |S| is the number of features in subset *S*;
- |N| is the total number of features;
- $f_{x}\left(S\right)$ is the output for the model using features in *S*;
- $f_{x}\left(S\cup \left\{j\right\}\right)$ is the model output using features in *S* along with feature *j*.

The difference $\left[f_{x}\left(S\cup \left\{j\right\}\right)-f_{x}\left(S\right)\right]$ quantifies the marginal contribution of feature *j* to the prediction when added to subset *S*. The factorial terms adjust for the different numbers of combinations in which *S* can occur with or without *j*, ensuring a fair attribution.

The SHAP summary plot is an indispensable tool in diabetes detection, offering a visual representation of the impact each feature holds over the prediction outcomes. It serves multiple functions: identifying critical features that significantly influence diabetes risk, thus guiding preventive and management strategies; illustrating the direction of impact; showing whether a particular factor like glucose level or BMI increases or decreases diabetes risk, which is vital for targeted medical interventions; and revealing the data distribution and potential outliers, aiding in the identification of anomalies or unusual cases that may require a further medical examination. By integrating SHAP summary plots into diabetes prediction models, healthcare professionals can enhance diagnostic accuracy, foster patient trust, and refine treatment approaches based on a transparent and interpretable foundation of machine learning predictions.

### 4.7. Ensemble Methods

Ensemble methods enhance the performance of different models by combining the outputs from several to improve prediction accuracy, particularly for complex tasks like diabetes prediction. These methods mitigate the weaknesses of individual models by aggregating their predictions in a way that reduces bias, variance, or both, leading to more robust and reliable outcomes.

Two prominent ensemble techniques are bagging and boosting:

- Bagging (Bootstrap Aggregating) trains more than one model independently on different subsets of the training data generated via random sampling with replacement. By averaging or voting over the predictions of these models, bagging reduces variance, thereby preventing overfitting and enhancing the model’s generalizability to unseen data.
- Boosting, on the other hand, sequentially trains models, where each subsequent model focuses on correcting the errors made by its predecessors. This iterative process reduces bias by gradually refining the prediction accuracy, making boosting highly effective for improving weak learners.

Both techniques are crucial for diabetes prediction models as they can handle high variability in patient data, capturing complex relationships between features while minimizing errors. By using ensemble methods, the overall prediction accuracy is significantly improved, which is critical for early detection and effective management of diabetes.

## 5. Experiment Details

This section elaborates on the design and execution of experiments performed in this study, followed by a presentation of the results and their systematic analysis. The performance of diabetes prediction models is evaluated using ten different metrics, providing insights into their robustness and effectiveness.

### 5.1. System Model

The system model for the diabetes prediction study, as depicted in Figure 1, begins with data pre-processing, where the diabetes dataset is cleaned and prepared. This is followed by dividing the dataset into training and testing with a ratio of 80:20 to ensure models learn from the majority and are validated on unseen data. Ten different machine learning algorithms are then applied to develop predictive models. These models include logistic regression, Gradient Boosting, XGBoost, AdaBoost, CatBoost, LightGBM, support vector machine, decision tree, random forest, and Multi-layer Neural Network. Following the initial training, Shapley Additive explanation (SHAP) summary plots are generated for each model to identify the contribution of individual features to the prediction outcomes. The top three most impactful features, as determined from the SHAP summaries, are selected for a focused evaluation of each model. Models then undergo hyperparameter tuning to optimize performance. A 3-fold cross-validation technique ensures robustness in model evaluation over limited data samples. In each fold, one subset is used for testing and the rest for training.

The evaluation of models is conducted using three sets of features: all features, the top three SHAP-selected features, and the remaining features, allowing an assessment of model performance under various conditions. The final stage involves a comprehensive performance evaluation using metrics such as MAE, MSE, and ROC_AUC, among others, to gauge the accuracy and consistency of each model in predicting diabetes.

### 5.2. Performance Metrics

This subsection details the performance metrics taken from [32,33] used to evaluate the implemented machine learning models predicting diabetes. The metrics include Mean Absolute Error (MAE), Mean Squared Error (MSE), receiver operating characteristic area under the curve (AUC), accuracy, precision, recall, F1-score, sensitivity, specificity, Logarithmic Loss (Logloss), Hamming Loss, and Kappa Score.

- **Mean Absolute Error (MAE) and Mean Squared Error (MSE):** These metrics measure the average magnitude of prediction errors, with MAE providing a linear score and MSE amplifying and severely penalizing large errors.
  (8) $$\begin{array}{cc} \mathrm{MAE} & =\frac{1}{n}\sum_{i=1}^{n}\left|y_{i}-{\hat{y}}_{i}\right|\\ \mathrm{MSE} & =\frac{1}{n}\sum_{i=1}^{n}{\left(y_{i}-{\hat{y}}_{i}\right)}^{2} \end{array}$$
  Here, $y_{i}$ is the actual value, ${\hat{y}}_{i}$ is the predicted value, and *n* is the number of observations.
- **Area Under the Curve (AUC):** This metric evaluates the model’s ability to discriminate between classes at various threshold settings. AUC is a comprehensive measure that considers all possible classification thresholds.
  (9) $$\mathrm{AUC}=\int_{0}^{1}\mathrm{TPR}\left(f\right)\;d\mathrm{FPR}\left(f\right)$$
  Here, TPR is the True Positive Rate (recall), and FPR is the False Positive Rate.
- **Accuracy (Acc):** This metric assesses the overall correctness of the model and is calculated as follows:
  (10) $$\mathrm{Acc}=\frac{TP+TN}{TP+TN+FP+FN}$$
  Here TP is true positives, TN is true negatives, FP is false positives, and FN is false negatives.
- **Precision (P) and Recall (R):** Precision measures the correctness achieved in positive prediction. At the same time, recall is the ability of the model to find all relevant cases (sensitivity).
  (11) $$\begin{array}{cc} P & =\frac{TP}{TP+FP}\\ R & =\frac{TP}{TP+FN} \end{array}$$
- **F1-Score ($F_{1}$):** Harmonic mean of precision and recall from (11), balancing the two in cases of uneven class distribution.
  (12) $$F_{1}=2\times \frac{P\times R}{P+R}$$
- **Sensitivity (Se) and Specificity (Sp):** Sensitivity (recall) measures the proportion of actual positives correctly identified, while specificity measures the proportion of negatives correctly identified.
  (13) $$\begin{array}{cc} \mathrm{Se} & =\frac{TP}{TP+FN}\\ \mathrm{Sp} & =\frac{TN}{TN+FP} \end{array}$$
- **Logloss (LL):** Measures the uncertainty of the model’s predictions based on how much the predicted probabilities deviate from the actual class labels.
  (14) $$\mathrm{LL}=-\frac{1}{n}\sum_{i=1}^{n}\left[y_{i}\log\left({\hat{y}}_{i}\right)+\left(1-y_{i}\right)\log\left(1-{\hat{y}}_{i}\right)\right]$$
  Here, $y_{i}$ is the actual label, ${\hat{y}}_{i}$ is the predicted probability for class 1, and *n* is the number of samples.
- **Hamming Loss (HL):** The fraction of incorrectly predicted labels indicates the error rate in multi-label classification.
  (15) $$\mathrm{HL}=\frac{1}{n}\sum_{i=1}^{n}⊮\left(y_{i}\neq {\hat{y}}_{i}\right)$$
  Here, ⊮ is the indicator function, which equals 1 when the true label $y_{i}$ differs from the predicted label ${\hat{y}}_{i}$, and *n* is the number of instances.
- **Kappa Score (K):** Measures the agreement of predictions with the actual classifications, adjusted for the possibility of the agreement occurring by chance.
  (16) $$K=\frac{P_{o}-P_{e}}{1-P_{e}}$$
  Here, $P_{o}$ is the observed agreement (the proportion of times the model’s predictions agree with the true labels), and $P_{e}$ is the expected agreement by chance.

These metrics provide a comprehensive framework for evaluating the performance of diabetes prediction models, facilitating the identification of the most effective model configurations.

## 6. Results and Discussions

The results are discussed in the following subsections, one for selecting Shapley Additive features and another for model evaluation. A comparison of machine learning algorithms for diabetes prediction uses ten different ML algorithms. The implementations use the Anaconda Navigator 2.5.4 with conda environment 24.4.0, an open-source integrated development environment, with the web-based, interactive Jupyter Notebook v7.0.8 on Visual Studio code v1.88.1, and operating under the Windows 11 system. Python v3.12.3 is the programming platform. Top influencing, less influencing, and combined datasets are employed separately as feature sets to assess the performance of the diabetes prediction system. A detailed comparison of these ML algorithms is provided, showcasing a systematic examination across various machine learning frameworks on the diabetes disease dataset.

### 6.1. Shapley Additive Features

**Figure 2:**
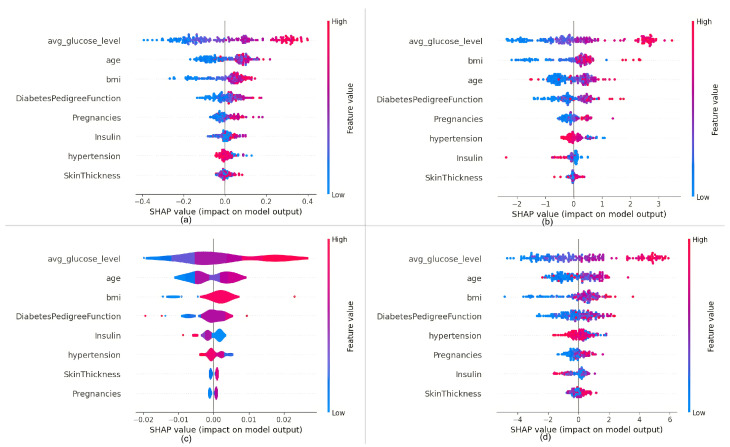
SHAP summary plots provide a comprehensive view of feature importance and contribution across different machine learning models: (**a**) random forest has the most contributing features reported as glucose level, age, and BMI; (**b**) Gradient Boost has the most contributing features reported as glucose level, BMI, and age; (**c**) Adaboost reports the most contributing features to be glucose level, age, and BMI; and (**d**) XGBoost reports the most contributing features to be glucose level, age, and BMI.

**Figure 3:**
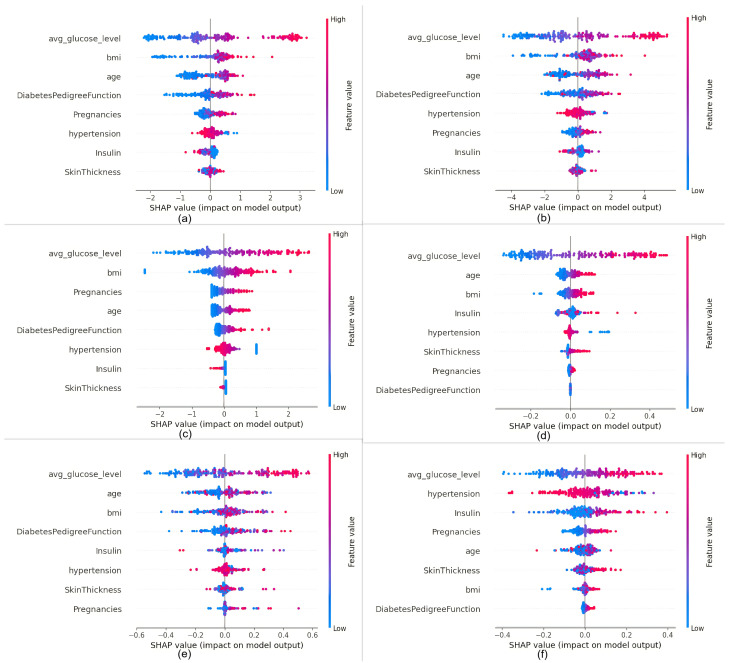
SHAP summary plots provide a comprehensive view of feature importance and contribution across different machine learning models: (**a**) CatBoost has the most contributing features reported as glucose level, BMI, and age; (**b**) LightGBM reports the most contributing features to be glucose level, BMI, and age; (**c**) logistic regression states the most contributing features to be glucose level, BMI, and pregnancies; (**d**) support vector machine reports the most contributing features to be glucose level, age, and BMI; (**e**) decision tree has the most contributing features reported as glucose level, age, and BMI; and (**f**) Multi-Layer Perceptron has the most contributing features reported as glucose level, hypertension, and insulin.

Initially, the SHAP explainer is generated for all the ten machine learning models as discussed in Section 5, providing SHAP values. From these SHAP values, the SHAP summary plot will be plotted. The most influencing features can be easily identified from each summary plot. The following Figure 2 and Figure 3 present the SHAP summary plots for all of these models.

Table 3 presents the three most influential features across ten models, as identified from their respective SHAP summary plots. It is noted that the feature ‘average glucose level’ consistently exerts the highest influence across all models. For models such as XGBoost, AdaBoost, support vector machine, decision tree, and random forest, the same top three features are observed—namely, average glucose level, age, and BMI—in this specific order. Conversely, models like Gradient Boosting, CatBoost, and LightGBM also share these top three features but in varied order: average glucose level, BMI, and age. Distinctively, the logistic regression model exhibits a different composition of the top three features, which include average glucose level, BMI, and Pregnancy. In contrast, the Multi-layer Neural Network model prioritizes average glucose level, hypertension, and insulin as the top three influencing features. This variance in the sets of top three features across different models underscores the diverse evaluative mechanisms employed in predicting diabetes.

### 6.2. Performance Comparison of Feature Sets

The performance of ten distinct machine learning models has been compared based on three different categorizations of feature sets, as delineated in Table 4, Table 5, Table 6, Table 7, Table 8, Table 9, Table 10, Table 11, Table 12 and Table 13.

These tables’ metrics (e.g., F1-score, recall, specificity, AUC, Logloss) are interdependent due to shared mathematical or probabilistic foundations. Changes in feature sets or decision thresholds can lead to correlated variations in these metrics, reflecting their interconnected behavior in evaluating model performance. Initially, a comprehensive set of all features is considered. Subsequently, the analysis narrows to only the top three most influential features identified via the SHAP summary plot. Lastly, a third set is evaluated, consisting exclusively of features other than the top three. The comparison of model performance across these feature sets is systematically presented in the referenced table, and the metrics symbols are as defined in Section 5.2.

It has been observed across various machine learning models that the utilization of all features generally enhances performance metrics such as Mean Absolute Error (MAE), Mean Squared Error (MSE), and area under the curve (AUC), as evidenced by data values in models like random forest and XGBoost (MAE, MSE, AUC of 0.16, 0.16, and 0.93, respectively for random forest with all features). Performance metrics are closely competitive when only the top three features are employed, especially in precision and recall (for instance, XGBoost shows a precision of 0.79 and a recall of 0.91 with the top three features), indicating the substantial influence of these select features. Conversely, a consistent reduction in performance is noted when employing features other than the top three, substantiating their comparatively lesser impact on model efficacy (as shown by a lower Kappa value of 0.24 in support vector machine with other features). This pattern highlights the critical role of a full feature set in achieving optimal model performance while also underscoring the effectiveness of key features in nearly matching the full set’s performance in certain models.

### 6.3. Performance Comparison of Various Learning Models

The meticulous evaluation of ten different machine learning models across three categories of feature sets, as detailed in Table 14, Table 15 and Table 16, reveals nuanced insights into their performances. The comprehensive inclusion of all features generally enhances the robustness of models, with LightGBM and random forest demonstrating superior performance metrics. Specifically, in Table 14, random forest achieves an exemplary AUC of 0.93, and LightGBM presents a promising accuracy and AUC of 0.82 and 0.89, respectively. These models maintain a commendable balance in precision and recall, with random forest reflecting an F1-score of 0.85.

Overall performance slightly changes across most models when only the top three features are utilized, as shown in Table 15. However, random forest, LightGBM, and XGBoost still perform robustly, achieving high AUC values of 0.89, 0.85, and 0.86, respectively, and maintaining substantial accuracy and F1-scores. This highlights their efficiency even with a limited set of features.

In scenarios where alternative features are exclusively employed (Table 16), random forest and LightGBM again stand out. Random forest delivers the highest AUC of 0.91, showcasing its ability to handle diverse feature sets effectively and maintain high classification accuracy. LightGBM also shows resilience, with strong performance metrics across the board, including an AUC of 0.87 and an accuracy of 0.78.

These observations underscore the adaptability and robust performance of the LightGBM and random forest models, irrespective of the feature set configuration, suggesting their suitability for various predictive modeling tasks within the diabetes dataset.

### 6.4. Performance Comparison of Ensemble Models

Based on the comprehensive analysis of performance metrics across different feature sets, it is determined that LightGBM and random forest emerge as strong candidates for inclusion in an ensemble model. LightGBM consistently demonstrated high-performance metrics across all feature subsets, indicating its stability and reliability in classification tasks. Similarly, superior performance across varying feature sets is exhibited by random forest, showcasing high AUC values, accuracy, precision, recall, and F1-score. Its robustness is evident even when trained on limited top features or other feature subsets. Combining LightGBM and random forest in an ensemble model can capitalize on their complementary strengths, and robustness in classification tasks can be ensured, potentially leading to improved overall performance.

In the performance comparison of ensemble models, as detailed in Table 17, the stacked Ensemble RF + LGBM and stacked Ensemble All models demonstrate remarkable efficacy across various metrics, highlighting their robustness in classification tasks. In this ensemble framework, RF and LGBM serve as base learners. Both models are independently optimized using grid search for hyperparameter tuning to maximize their performance. Once optimized, these models are integrated into a unified predictive system, where their outputs (class probabilities) are combined. The soft voting mechanism [34] aggregates the predicted probabilities for each class from RF and LGBM. The class with the highest average probability across the two models is then selected as the final prediction. The stacked Ensemble RF+LGBM model, which combines random forest and LightGBM through a stacking ensemble approach, achieves the highest AUC of 0.93, with an accuracy of 0.84, precision of 0.80, and a recall of 0.92. This ensemble also shows a superior F1-score of 0.86, a sensitivity of 0.92, and a specificity of 0.77, indicating a balanced precision–recall trade-off and strong discriminative ability. Moreover, it reports the lowest Logloss of 0.36 and a Kappa of 0.69, suggesting high agreement with true classifications. The Ensemble All model also performs commendably, closely matching the Ensemble RF+LGBM in most metrics but slightly lower in AUC (0.91), precision (0.79), and specificity (0.76). These observations affirm that integrating random forest and LightGBM into a single ensemble model leverages their strengths and enhances the overall predictive performance, making it a potent approach in machine learning tasks.

It is important to recognize that a performance comparison of ensemble models using either the top three most influential features or other less influential features is not feasible, as each model harbors a unique set of the top three most influential features. This limitation indicates the specific configurations and feature dependencies intrinsic to each model, underscoring the customized approach in their application and evaluation.

Interestingly, in this study, the models chosen for ensembling, namely LightGBM and random forest, share the same top three most influential features, although their order varies, as demonstrated in Table 3. Improved performance metrics are observed in Table 18 where only the top three most influential features are considered. The ensemble of all models remains unfeasible due to the lack of uniformity in the top most influential features across models. However, the combined Ensemble RF + LGBM model, which integrates the strengths of random forest and LightGBM, achieves the highest area under the curve of 0.94, with notable accuracy of 0.86, precision of 0.82, and recall of 0.92. This ensemble exhibits a superior F1-score of 0.87, sensitivity of 0.92, and specificity of 0.8. The results achieved by the Ensemble RF + LGBM model for the top three features are compared with those of existing works in Table 19, showcasing its comparative advantage.

In the comparative analysis presented in Table 19, the performance metrics of various approaches to the same problem are illustrated, underscoring the superiority of the method proposed by this current work. The highest area under the curve is achieved by our approach, recorded at 0.9424, indicating an exceptional ability to distinguish between classes. Moreover, superior performance is exhibited in terms of recall (R=0.9208) and $F_{1}$-score ($F_{1}=0.8691$), which are critical measures that reflect accuracy and the balance between precision and recall. Sensitivity superiority is also demonstrated, with values of 0.9208, suggesting robustness in the correct identification of true positives and true negatives. This comprehensive superiority in performance establishes the method proposed by our team as a more effective and reliable solution than the existing methodologies. All methods compared in Table 19 utilized the PIMA Indian Diabetes Dataset with consistent 80-20 data splitting, standard pre-processing, identical evaluation metrics, and a similar computational setup to ensure uniformity and comparability.

## 7. Conclusions

In the research, an exhaustive evaluation of ten diverse machine learning models has been meticulously carried out using three distinct categories of feature sets on a diabetes dataset. The results indicate that adopting the top three most influential features identified through Shapley Additive explanations can significantly optimize the performance of ensemble machine learning models in diabetes prediction. For instance, when using the top three features, the ensemble model achieved an AUC of 0.94 and an accuracy of 0.86, compared to its performance with all features, where it reached an area under the curve of 0.93 and accuracy of0.84. This suggests a minimal trade-off between feature reduction for computational efficiency and maintaining high predictive accuracy. It adds to existing research by demonstrating how targeted feature selection based on Shapley values can optimize model efficiency without significantly compromising accuracy. It provides a practical approach for feature reduction in complex predictive tasks. This approach aids in efficiently deploying predictive models in clinical settings, where quick and accurate decision-making is crucial.

## Figures and Tables

**Table 1:** Attribute information of diabetes dataset.

Attribute	Description	Range
Pregnancies	Number of pregnancies experienced by the individual	0–17
Avg glucose level	Average plasma glucose concentration after 2 h of an oral glucose test	0–199
Hypertension	Diastolic blood pressure measured in millimeters of mercury (mm Hg)	0–122
Skin Thickness	Thickness of the triceps skin fold measured in millimeters (mm)	0–99
Insulin	Serum insulin level measured 2 h post-test (mu U/mL)	0–846
BMI	Body Mass Index (BMI), calculated as weight in kg divided by height squared	0–67.1
Diabetes pedigree function	A function that estimates diabetes likelihood based on family history	0.08–2.42
Age	Age of the individual in years	21–81
Outcome	Diagnosis outcome (0 = no diabetes, 1 = diabetes)	0/1

**Table 2:** Features statistics of diabetes dataset.

Attributes	Count	Mean	Standard Deviation	min	25%	50%	75%	max
Pregnancies	768	3.85	3.37	0	1	3	6	17
Avg glucose level	768	120.89	31.97	0	99	117	140.25	199
Hypertension	768	69.11	19.36	0	62	72	80	122
Skin thickness	768	20.54	15.95	0	0	23	32	99
Insulin	768	79.80	115.24	0	0	30.50	127.25	846
BMI	768	31.99	7.88	0	27.30	32	36.60	67.10
Diabetes pedigree function	768	0.47	0.33	0.08	0.24	0.37	0.63	2.42
Age	768	33.24	11.76	21	24	29	41	81

**Table 3:** Top 3 most influencing Shapley Additive Features for each model.

Model	Top 3 Features
Logistic Regression	avg_glucose_level, BMI, Pregnancies
Gradient Boosting	avg_glucose_level, BMI, age
XGBoost	avg_glucose_level, age, BMI
AdaBoost	avg_glucose_level, age, BMI
CatBoost	avg_glucose_level, BMI, age
LightGBM	avg_glucose_level, BMI, age
Support Vector Machine	avg_glucose_level, age, BMI
Decision Tree	avg_glucose_level, age, BMI
Random Forest	avg_glucose_level, age, BMI
Multi-layered Neural Network	avg_glucose_level, hypertension, Insulin

**Table 4:** Performance comparison on logistic regression with all categories of feature sets.

Feature Types	MAE	MSE	AUC	Acc	P	R	$F_{1}$	Se	Sp	LL	HL	K
All Features	0.23	0.23	0.84	0.77	0.75	0.81	0.78	0.81	0.73	0.49	0.23	0.54
Top 3 Features	0.22	0.22	0.84	0.78	0.77	0.78	0.78	0.78	0.77	0.5	0.22	0.55
Other Features	0.32	0.32	0.7	0.68	0.69	0.66	0.68	0.66	0.7	0.63	0.32	0.36

**Table 5:** Performance comparison on Gradient Boosting with all categories of feature sets.

Feature Types	MAE	MSE	AUC	Acc	P	R	$F_{1}$	Se	Sp	LL	HL	K
All Features	0.2	0.2	0.88	0.8	0.76	0.89	0.82	0.89	0.71	0.46	0.2	0.6
Top 3 Features	0.18	0.18	0.88	0.82	0.77	0.91	0.84	0.91	0.73	0.45	0.18	0.64
Other Features	0.3	0.3	0.8	0.7	0.69	0.75	0.72	0.75	0.66	0.55	0.3	0.41

**Table 6:** Performance comparison on AdaBoost with all categories of feature sets.

Feature Types	MAE	MSE	AUC	Acc	P	R	$F_{1}$	Se	Sp	LL	HL	K
All Features	0.24	0.24	0.82	0.76	0.75	0.8	0.78	0.8	0.73	0.66	0.24	0.53
Top 3 Features	0.24	0.24	0.81	0.76	0.73	0.82	0.77	0.82	0.69	0.65	0.24	0.51
Other Features	0.35	0.35	0.73	0.65	0.64	0.7	0.67	0.7	0.6	0.68	0.35	0.3

**Table 7:** Performance comparison on random forest with all categories of feature sets.

Feature Types	MAE	MSE	AUC	Acc	P	R	$F_{1}$	Se	Sp	LL	HL	K
All Features	0.16	0.16	0.93	0.84	0.79	0.92	0.85	0.92	0.76	0.36	0.16	0.68
Top 3 Features	0.18	0.18	0.89	0.82	0.76	0.92	0.83	0.92	0.71	0.6	0.18	0.63
Other Features	0.2	0.2	0.91	0.8	0.77	0.87	0.82	0.87	0.74	0.41	0.2	0.61

**Table 8:** Performance comparison on XGBoost with all categories of feature sets.

Feature Types	MAE	MSE	AUC	Acc	P	R	$F_{1}$	Se	Sp	LL	HL	K
All Features	0.18	0.18	0.89	0.82	0.78	0.91	0.84	0.91	0.74	0.5	0.18	0.65
Top 3 Features	0.17	0.17	0.86	0.83	0.79	0.91	0.84	0.91	0.75	0.52	0.17	0.66
Other Features	0.2	0.2	0.86	0.8	0.76	0.89	0.82	0.89	0.71	0.49	0.2	0.6

**Table 9:** Performance comparison on CatBoost with all categories of feature sets.

Feature Types	MAE	MSE	AUC	Acc	P	R	$F_{1}$	Se	Sp	LL	HL	K
All Features	0.17	0.17	0.9	0.83	0.78	0.92	0.85	0.92	0.74	0.42	0.17	0.66
Top 3 Features	0.19	0.19	0.87	0.81	0.76	0.92	0.83	0.92	0.7	0.47	0.19	0.62
Other Features	0.22	0.22	0.85	0.78	0.75	0.86	0.8	0.86	0.71	0.49	0.22	0.57

**Table 10:** Performance comparison on LightGBM with all categories of feature sets.

Feature Types	MAE	MSE	AUC	Acc	P	R	$F_{1}$	Se	Sp	LL	HL	K
All Features	0.18	0.18	0.89	0.82	0.78	0.9	0.84	0.9	0.75	0.49	0.18	0.65
Top 3 Features	0.19	0.19	0.85	0.81	0.77	0.89	0.83	0.89	0.73	0.56	0.19	0.62
Other Features	0.22	0.22	0.87	0.78	0.74	0.87	0.8	0.87	0.69	0.56	0.22	0.56

**Table 11:** Performance comparison on support vector machine with all categories of feature sets.

Feature Types	MAE	MSE	AUC	Acc	P	R	$F_{1}$	Se	Sp	LL	HL	K
All Features	0.24	0.24	0.84	0.76	0.75	0.81	0.78	0.81	0.72	0.49	0.24	0.53
Top 3 Features	0.28	0.28	0.8	0.72	0.74	0.69	0.71	0.69	0.75	0.56	0.28	0.44
Other Features	0.38	0.38	0.71	0.62	0.62	0.65	0.63	0.65	0.59	0.62	0.38	0.24

**Table 12:** Performance comparison on decision tree with all categories of feature sets.

Feature Types	MAE	MSE	AUC	Acc	P	R	$F_{1}$	Se	Sp	LL	HL	K
All Features	0.17	0.17	0.83	0.83	0.79	0.9	0.84	0.9	0.76	6.13	0.17	0.66
Top 3 Features	0.18	0.18	0.82	0.82	0.79	0.87	0.83	0.87	0.77	6.49	0.18	0.64
Other Features	0.24	0.24	0.76	0.76	0.75	0.81	0.78	0.81	0.72	8.47	0.24	0.53

**Table 13:** Performance comparison on Multi-layered Neural Network with all categories of feature sets.

Feature Types	MAE	MSE	AUC	Acc	P	R	$F_{1}$	Se	Sp	LL	HL	K
All Features	0.26	0.26	0.78	0.74	0.74	0.74	0.74	0.74	0.74	0.57	0.26	0.48
Top 3 Features	0.4	0.4	0.63	0.6	0.61	0.59	0.6	0.59	0.62	0.69	0.4	0.21
Other Features	0.35	0.35	0.74	0.65	0.63	0.74	0.68	0.74	0.56	0.61	0.35	0.3

**Table 14:** Comprehensive performance evaluation of learning models on full-feature diabetes dataset.

Metrics	Logistic Reg.	Gradient Boost	XGBoost	AdaBoost	CatBoost	LightGBM	Support Vector Machine	Decision Tree	Random Forest	Multi-Layer Neural
MAE	0.23	0.2	0.18	0.24	0.17	0.18	0.24	0.17	0.16	0.26
MSE	0.23	0.2	0.18	0.24	0.17	0.18	0.24	0.17	0.16	0.26
AUC	0.84	0.88	0.89	0.82	0.9	0.89	0.84	0.83	0.93	0.78
Acc	0.77	0.8	0.82	0.76	0.83	0.82	0.76	0.83	0.84	0.74
P	0.75	0.76	0.78	0.75	0.78	0.78	0.75	0.79	0.79	0.74
R	0.81	0.89	0.91	0.8	0.92	0.9	0.81	0.9	0.92	0.74
$F_{1}$	0.78	0.82	0.84	0.78	0.85	0.84	0.78	0.84	0.85	0.74
Se	0.81	0.89	0.91	0.8	0.92	0.9	0.81	0.9	0.92	0.74
Sp	0.73	0.71	0.74	0.73	0.74	0.75	0.72	0.76	0.76	0.74
LL	0.49	0.46	0.5	0.66	0.42	0.49	0.49	6.13	0.36	0.57
HL	0.23	0.2	0.18	0.24	0.17	0.18	0.24	0.17	0.16	0.26
K	0.54	0.6	0.65	0.53	0.66	0.65	0.53	0.66	0.68	0.48

**Table 15:** Comprehensive performance evaluation of learning models on full-feature diabetes dataset only for top three features.

Metrics	Logistic Reg.	Gradient Boost	XGBoost	AdaBoost	CatBoost	LightGBM	Support Vector Machine	Decision Tree	Random Forest	Multi-Layer Neural
MAE	0.22	0.18	0.17	0.24	0.19	0.19	0.28	0.18	0.18	0.4
MSE	0.22	0.18	0.17	0.24	0.19	0.19	0.28	0.18	0.18	0.4
AUC	0.84	0.88	0.86	0.81	0.87	0.85	0.8	0.82	0.89	0.63
Acc	0.78	0.82	0.83	0.76	0.81	0.81	0.72	0.82	0.82	0.6
P	0.77	0.77	0.79	0.73	0.76	0.77	0.74	0.79	0.76	0.61
R	0.78	0.91	0.91	0.82	0.92	0.89	0.69	0.87	0.92	0.59
F1	0.78	0.84	0.84	0.77	0.83	0.83	0.71	0.83	0.83	0.6
Se	0.78	0.91	0.91	0.82	0.92	0.89	0.69	0.87	0.92	0.59
Sp	0.77	0.73	0.75	0.69	0.7	0.73	0.75	0.77	0.71	0.62
LL	0.5	0.45	0.52	0.65	0.47	0.56	0.56	6.49	0.6	0.69
HL	0.22	0.18	0.17	0.24	0.19	0.19	0.28	0.18	0.18	0.4
K	0.55	0.64	0.66	0.51	0.62	0.62	0.44	0.64	0.63	0.21

**Table 16:** Comprehensive performance evaluation of learning models on full-feature diabetes dataset other than top three features.

Metrics	Logistic Reg.	Gradient Boost	XGBoost	AdaBoost	CatBoost	LightGBM	Support Vector Machine	Decision Tree	Random Forest	Multi-Layer Neural
MAE	0.32	0.3	0.2	0.35	0.22	0.22	0.38	0.24	0.2	0.35
MSE	0.32	0.3	0.2	0.35	0.22	0.22	0.38	0.24	0.2	0.35
AUC	0.7	0.8	0.86	0.73	0.85	0.87	0.71	0.76	0.91	0.74
Acc	0.68	0.7	0.8	0.65	0.78	0.78	0.62	0.76	0.8	0.65
P	0.69	0.69	0.76	0.64	0.75	0.74	0.62	0.75	0.77	0.63
R	0.66	0.75	0.89	0.7	0.86	0.87	0.65	0.81	0.87	0.74
$F_{1}$	0.68	0.72	0.82	0.67	0.8	0.8	0.63	0.78	0.82	0.68
Se	0.66	0.75	0.89	0.7	0.86	0.87	0.65	0.81	0.87	0.74
Sp	0.7	0.66	0.71	0.6	0.71	0.69	0.59	0.72	0.74	0.56
LL	0.63	0.55	0.49	0.68	0.49	0.56	0.62	8.47	0.41	0.61
HL	0.32	0.3	0.2	0.35	0.22	0.22	0.38	0.24	0.2	0.35
K	0.36	0.41	0.6	0.3	0.57	0.56	0.24	0.53	0.61	0.3

**Table 17:** Comprehensive performance evaluation of ensemble learning models on full-feature diabetes dataset.

Models	MAE	MSE	AUC	Acc	P	R	$F_{1}$	Se	Sp	LL	HL	K
Ensemble All	0.16	0.16	0.91	0.84	0.79	0.92	0.85	0.92	0.76	0.4	0.16	0.68
Ensemble RF + LGBM	0.16	0.16	0.93	0.84	0.8	0.92	0.86	0.92	0.77	0.36	0.16	0.69
Random Forest	0.16	0.16	0.93	0.84	0.79	0.92	0.85	0.92	0.76	0.36	0.16	0.68
LightGBM	0.18	0.18	0.89	0.82	0.78	0.9	0.84	0.9	0.75	0.49	0.18	0.65

**Table 18:** Performance comparison of ensemble models with only top 3 features.

Models	MAE	MSE	AUC	Acc	P	R	$F_{1}$	Se	Sp	LL	HL	K
Ensemble RF + LGBM	0.15	0.15	0.94	0.86	0.82	0.92	0.87	0.92	0.8	0.35	0.14	0.65
Random Forest	0.18	0.18	0.89	0.82	0.76	0.92	0.83	0.92	0.71	0.6	0.18	0.63
LightGBM	0.19	0.19	0.85	0.81	0.77	0.89	0.83	0.89	0.73	0.56	0.19	0.62

**Table 19:** Performance comparison of equivalent approaches.

Approaches	AUC	Acc	P	R	$F_{1}$	Se	Sp
Belsti Yitayeh et al. [10]	0.93	0.85	**0.90**	0.78	0.84	0.81	**0.90**
Chang et al. [17]	0.8624	0.7987	0.894	NA	0.8517	0.8843	0.75
Sharma et al. [14]	NA	**0.9057**	NA	NA	0.7503	0.8224	0.7323
Dharmarathne et al. [24]	NA	0.76–0.8	0.89	0.5–0.76	NA	NA	NA
proposed approach	**0.9424**	0.8606	0.823	**0.9208**	**0.8691**	**0.9208**	0.80

NA—data not available for the reference.

## Data Availability

The Pima Indians Diabetes Dataset is available online at https://www.kaggle.com/datasets/uciml/pima-indians-diabetes-database/data (accessed on 31 October 2024).
